# CT-based radiomics models predict spontaneous intracerebral hemorrhage expansion and are comparable with CT angiography spot sign

**DOI:** 10.3389/fneur.2024.1332509

**Published:** 2024-02-26

**Authors:** Qingrun Li, Feng Li, Hao Liu, Yan Li, Hongri Chen, Wenrui Yang, Shaofeng Duan, Hongying Zhang

**Affiliations:** ^1^Department of Radiology, Traditional Chinese Medicine Hospital of Dianjiang Chongqing, Chongqing, China; ^2^Department of Research and Development, Yizhun Medical AI Co. Ltd., Beijing, China; ^3^Department of Radiology, Northern Jiangsu People's Hospital, Yangzhou, Jiangsu, China; ^4^Precision Health Institution, GE Healthcare, Shanghai, China

**Keywords:** intracerebral hemorrhage, hematoma expansion, radiomics, computed tomography, spot sign

## Abstract

**Background and purpose:**

This study aimed to investigate the efficacy of radiomics, based on non-contrast computed tomography (NCCT) and computed tomography angiography (CTA) images, in predicting early hematoma expansion (HE) in patients with spontaneous intracerebral hemorrhage (SICH). Additionally, the predictive performance of these models was compared with that of the established CTA spot sign.

**Materials and methods:**

A retrospective analysis was conducted using CT images from 182 patients with SICH. Data from the patients were divided into a training set (145 cases) and a testing set (37 cases) using random stratified sampling. Two radiomics models were constructed by combining quantitative features extracted from NCCT images (the NCCT model) and CTA images (the CTA model) using a logistic regression (LR) classifier. Additionally, a univariate LR model based on the CTA spot sign (the spot sign model) was established. The predictive performance of the two radiomics models and the spot sign model was compared according to the area under the receiver operating characteristic (ROC) curve (AUC).

**Results:**

For the training set, the AUCs of the NCCT, CTA, and spot sign models were 0.938, 0.904, and 0.726, respectively. Both the NCCT and CTA models demonstrated superior predictive performance compared to the spot sign model (all *P* < 0.001), with the performance of the two radiomics models being comparable (*P* = 0.068). For the testing set, the AUCs of the NCCT, CTA, and spot sign models were 0.925, 0.873, and 0.720, respectively, with only the NCCT model exhibiting significantly greater predictive value than the spot sign model (*P* = 0.041).

**Conclusion:**

Radiomics models based on NCCT and CTA images effectively predicted HE in patients with SICH. The predictive performances of the NCCT and CTA models were similar, with the NCCT model outperforming the spot sign model. These findings suggest that this approach has the potential to reduce the need for CTA examinations, thereby reducing radiation exposure and the use of contrast agents in future practice for the purpose of predicting hematoma expansion.

## Introduction

Spontaneous intracerebral hemorrhage (SICH) is a prevalent subtype of stroke, accounting for ~10%−15% of all strokes. Unlike ischemic stroke, SICH leads to more severe disability and higher mortality rates, with nearly 40% mortality within the first month (1). Early hematoma expansion (HE) occurs in ~30% of SICH patients and is strongly associated with unfavorable outcomes (2). Studies have shown that, for every 1 mL increase in bleeding, the risk of death or disability increases by ~5% (3). Therefore, the accurate identification of patients at risk of HE is crucial in clinical settings.

Several imaging markers have been proven to serve as reliable predictors for determining HE: these include an irregular shape, the island sign, hypodensities within the hematoma, the blend sign, the black hole sign, and the swirl sign on non-contrast computed tomography (NCCT), as well as the spot sign on computed tomography angiography (CTA) (4–10). In particular, the CTA spot sign has been widely adopted as a benchmark for prediction of HE in clinical practice. However, these markers are susceptible to subjective interpretation influenced by the researcher's experience, and many lack sufficient sensitivity. For example, despite the promising performance of the CTA spot sign, the pooled sensitivity values reported in three previous meta-analyses are only 0.53, 0.62, and 0.57 (11–13). In other words, these predictors are suboptimal for accurately predicting HE. Therefore, in this study, we aimed to explore a more sensitive, objective, and convenient approach.

Radiomics, an emerging field of research, utilizes data mining algorithms to extract quantitative features from medical images (14). It has garnered significant attention in oncological investigations (15, 16). Recently, researchers have explored the potential of radiomics in predicting the expansion of intracerebral hemorrhage (17–19). Their studies have demonstrated the effectiveness of radiomics models in predicting HE, surpassing conventional radiological and clinical models. However, these previous studies have focused solely on NCCT data. In clinical practice, multimodal CT images, such as CTA images, are available for patient evaluation. CTA images not only reveal hidden vascular information within the hemorrhage but also enhance changes in the image construct. Considering these advantages, we hypothesized that a radiomics model based on CTA images would outperform models based on other image types. Therefore, in this study, we aimed to develop separate radiomics models based on NCCT and CTA images to predict HE. Additionally, we aimed to evaluate their predictive performance by comparing them with the established spot sign.

## Materials and methods

### Patients

**Figure 1:**
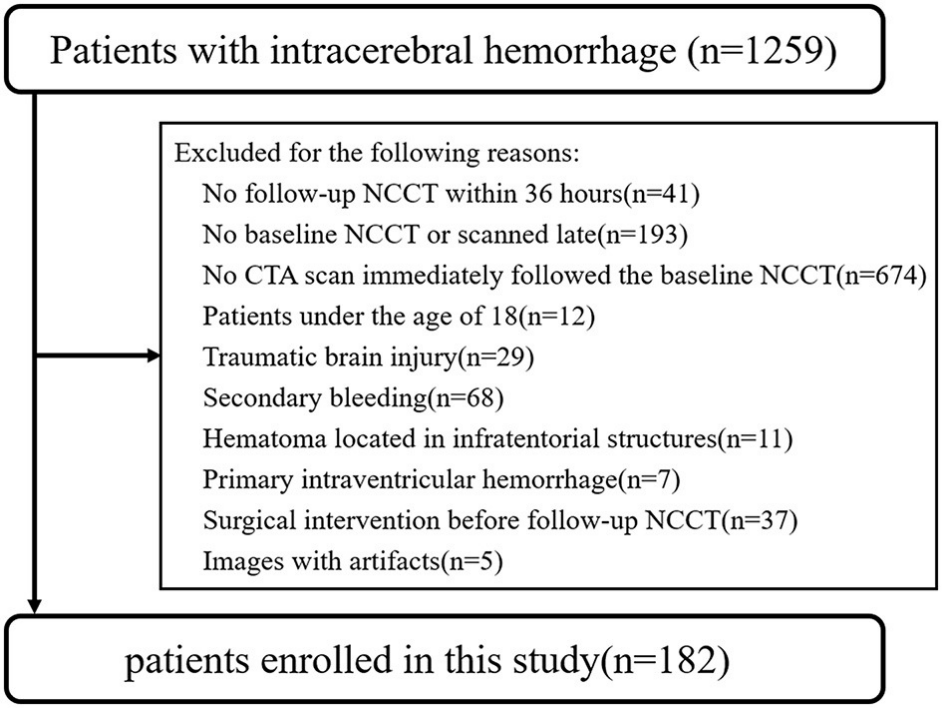
Flowchart of patient enrolment and exclusion criteria.

This retrospective study enrolled patients with SICH who were admitted to Northern Jiangsu People's Hospital via the emergency department between December 2015 and December 2020. Patients eligible to participate were those with SICH aged 18 years or older who underwent initial NCCT followed by cranial CTA within 6 h of symptom onset and follow-up NCCT within 36 h. Patients with traumatic brain injury, secondary intracerebral hemorrhage resulting from an aneurysm, vascular malformation, brain tumor, or hemorrhagic transformation of infarction, as well as those with infratentorial hematoma or primary intraventricular hemorrhage, those who underwent surgical intervention before follow-up NCCT, and those with CT images with artifact, were excluded from the study. A flowchart illustrating the patient selection process is shown in Figure 1.

This retrospective study was approved by the hospital's ethics committee, and the requirement for informed consent was waived.

### Image acquisition

Image acquisition was performed using a 64-row, 128-slice scanner (Optima CT660, GE Healthcare, Chicago, IL, USA) and an 80-row, 160-slice scanner (uCT 780, UIH, Shanghai, China). The scanning protocols for the Optima CT660 scanner consisted of a tube voltage of 120kV, automatic tube current, a collimation width of 64 mm, a scanning field of 250 mm, and slice thickness and interslice spacing of 5 mm and 0.625 mm for NCCT and CTA, respectively. For the uCT 780 scanner, the scanning protocols consisted of a tube voltage of 120kV, automatic tube current, a collimation width of 40 mm, a scanning field of 300 mm, and slice thickness and interslice spacing of 5 mm and 0.5 mm for NCCT and CTA, respectively. The scanning range extended from the base to the top of the skull. Test bolus technology was utilized to determine the appropriate CTA acquisition time. During CTA, 50–70 mL of iodixanol (Xiansu, Yangtze River Pharmaceutical Co, Ltd, Jiangsu, China; 320 mg I/mL) was intravenously injected at a rate of 5 mL/s via a power injector through the antecubital vein. All images were transferred to the post-processing workstation AW4.7 (GE Healthcare, USA).

### Radiological analysis

Automated hematoma recognition was performed on initial and follow-up CT images using the Stroke VCAR software package on the AW4.7 workstation; this assisted in segmenting hematoma areas and measuring hematoma volume. In this study, HE was defined as an increase in hematoma volume of ≥ 6 mL or ≥ 33% on follow-up CT compared to initial CT (20). Based on this criterion, all patients were categorized as either HE or non-HE. For SICH patients with intraventricular hemorrhage extension, the classification was independently verified by two physicians, one with 3 years and the other with 15 years of experience in radiodiagnosis, and their determinations were found to be consistent.

To evaluate the presence of the CTA spot sign in CTA images, two other neuroimaging diagnostic physicians, one with 2 years and the other with 20 years of experience, conducted independent assessments. Any discrepancies were resolved through joint discussion to reach a consensus. Both readers were blinded to all clinical information. Subsequently, a binary logistic regression (LR) model for the CTA spot sign (the spot sign model) was developed. The assessment included evaluating the location, shape, intraventricular hemorrhage extension, swirl sign, blend sign, black hole sign, and island sign, all of which were documented. Hematoma locations were classified as lobar or deep (involving the basal ganglia and/or thalamus) based on the location of the main body of the hematoma. The shape of the hematoma was recorded as either irregular or regular (5).

### Radiomics analysis

#### Lesion segmentation

**Figure 2:**
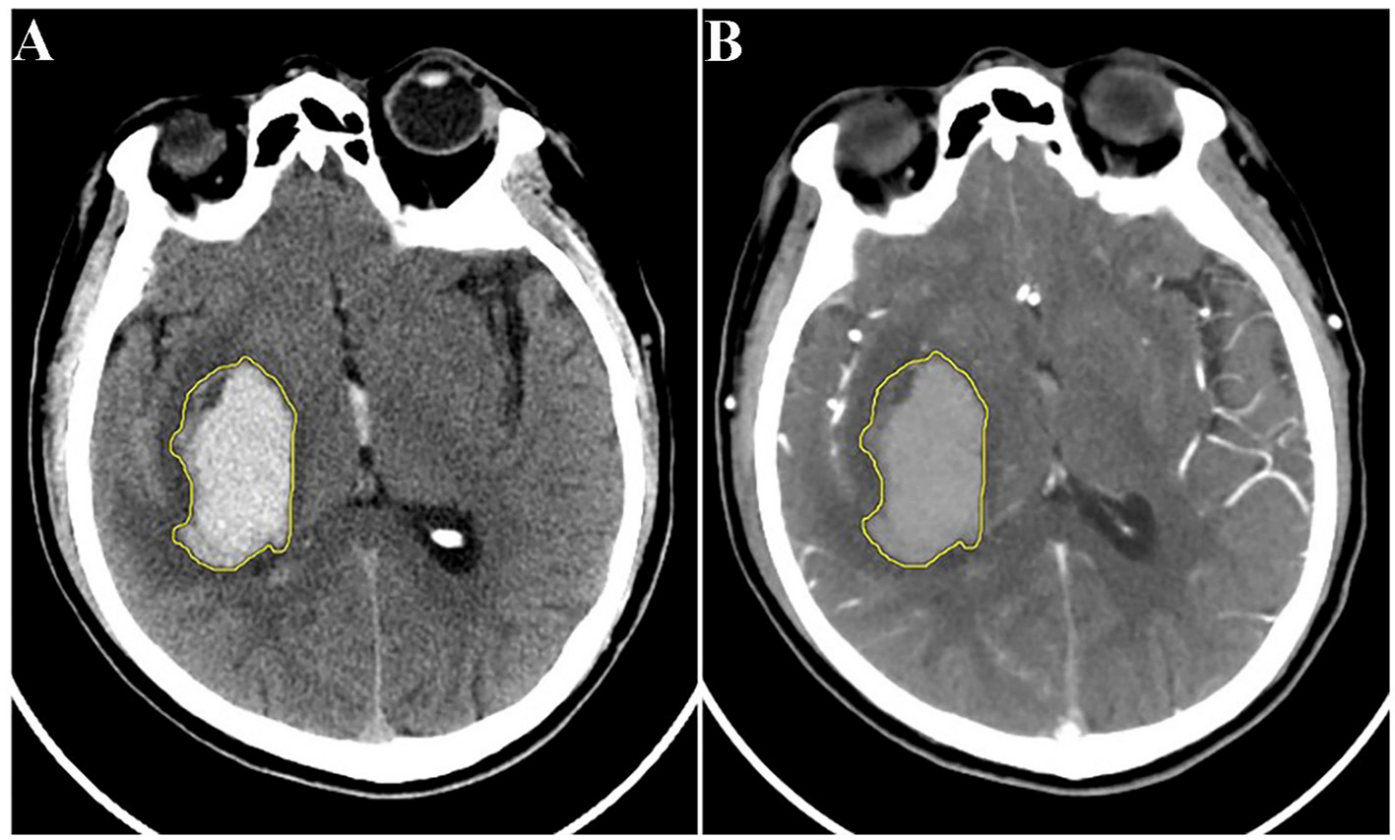
Schematic diagram of hematoma segmentation: **(A)** NCCT image; **(B)** reconstructed CTA image of the same patient. CTA, computed tomography angiography; NCCT, non-contrast computed tomography.

To mitigate the influence of varying slice thickness and interslice spacing across different CT scanners, as well as the distinctions between CTA and NCCT images, all original CTA images were reconstructed with a consistent slice thickness and interslice spacing of 5 mm, matching that of the NCCT images. Subsequently, both the NCCT and the reconstructed CTA images for the enrolled patients were exported in DICOM format and transferred to the DARWIN intelligent research platform (Yizhun Medical AI technology, Beijing, China, https://arxiv.org/abs/2009.00908). A volume of interest (VOI) for the hematoma was manually delineated and segmented layer by layer in the NCCT images, following the boundary of the hematoma from top to bottom. This delineation was then applied to the CTA images, with necessary adjustments made to derive the tailored VOI (Figure 2). Segmentation of the hematoma VOIs was independently carried out by the aforementioned two neuroimaging diagnosticians.

### Feature extraction and selection

A total of 120 quantitative features were extracted from each VOI in the original images using the pyradiomics package (http://pyradiomics.readthedocs.io/en/latest/index.html). In addition, seven filters were employed to transform the original images to capture additional information. These filters included the exponential filter, gradient filter, local binary pattern filter, logarithm filter, square filter, square root filter, and wavelet filter. Collectively, these processes resulted in the extraction of 1,688 candidate features. The candidate features were then categorized into three groups based on their relevance to (1) shape, (2) first-order statistics (histogram features), and (3) second-order statistics (texture features). The intraclass correlation coefficient (ICC) was computed to assess the reproducibility of feature extraction. Only features with an ICC >0.75 were included for further analysis.

To address the issue of redundant features, ANOVA *F*-test statistic was employed to select the top 100 features. Prior to feature screening, all features were standardized using Z-scores. The selected features were sorted based on their F-values, with higher values indicating lower *p*-values. Subsequently, the least absolute shrinkage and selection operation (LASSO) LR algorithm was employed to further reduce data redundancy and identify stable features through the use of non-zero coefficients. To ensure an unbiased comparison between the two models, an equal number of features were also selected using LASSO. Considering the relationship between the sample size and the number of features, we set this number at 10 on empirical grounds.

### Model construction

To ensure the integrity of the data distribution and minimize the introduction of bias during data processing, data from all patients were randomly stratified into a training set and a testing set in a ratio of 4:1. This approach maintained the consistency of data distribution between the two sets.

The features selected from both NCCT and CTA images were utilized to train the radiomics prediction models in conjunction with the widely used and effective LR machine learning classifier (21). The prediction capabilities of the constructed models were subsequently evaluated using an independent testing set. The predictive performances of the two radiomics models (the NCCT model and CTA model) were then compared with that of the spot sign model.

### Statistical analysis

An independent samples *t*-test or Mann–Whitney *U-*test was employed for continuous variables, and the chi-squared test was adopted for categorical variables. Continuous variables are reported in the form of mean ± standard deviation, and categorical variables are summarized in the form of count (percentage). The predictive performance of each model in estimating hematoma enlargement was evaluated via receiver operating characteristic (ROC) curve analysis. The area under the curve (AUC) values of the ROC curves were compared using the DeLong test. Statistical significance was determined when the bilateral *p* < 0.05. All statistical analyses were conducted using the SPSS software package (version 25.0), and the MedCalc software package (version 18.11.3) was utilized to generate and compare the ROC curves.

## Results

### Patient characteristics

Following the aforementioned criteria, 182 patients diagnosed with SICH were included in this study. Based on the follow-up CT, these patients were categorized into the HE group (67 cases) or the non-HE group (115 cases). Table 1 presents the statistical analysis of relevant factors, revealing a significant difference between the HE group (4 cases) and the non-HE group (0 cases) in terms of the pre-onset use of anticoagulants (warfarin) (*P* = 0.017). However, no significant differences between the two groups were observed in terms of gender, age, systolic blood pressure, diastolic blood pressure, or the use of antiplatelet drugs (aspirin) (all *P* > 0.05).

**Table 1:** Comparison of demographic, clinical, and baseline radiological characteristics between the HE group and the non-HE group.

	**HE group (*n =* 67)**	**non-HE group (*n =* 115)**	***p*-value**
Gender (male/female)	45/22	73/42	0.615^#^
Age (y)	60.6 ± 13.0	59.1 ± 13.5	0.466^*^
Systolic blood pressure (mmHg)	160.2 ± 25.2	160.3 ± 24.5	0.968^*^
Diastolic blood pressure (mmHg)	91.4 ± 16.6	90.4 ± 14.1	0.685^*^
Use of anticoagulants (warfarin)	4 (6.0)	0 (0)	**0.017** ^#^
Symptom onset to baseline CT (h)	3.17 ± 1.28	3.47 ± 1.37	0.226^*^
Use of antiplatelets (aspirin)	5 (7.5)	3 (2.6)	0.244^#^
Location (deep/lobe)	39/28	82/33	0.071^#^
Intraventricular hemorrhage extension	15 (22.4)	20 (22.1)	0.409^#^
Initial volume (mL)	43.3 ± 24.9	27.5 ± 17.1	<0.001^*^
Shape, irregular	38 (56.7)	42 (36.5)	**0.008** ^#^
Swirl sign	26 (38.8)	24 (20.9)	**0.009** ^#^
Blend sign	24 (35.8)	15 (13.0)	<**0.001**^#^
Black hole sign	9 (13.4)	5 (4.3)	**0.027** ^#^
Island sign	14 (20.9)	9 (7.8)	**0.010** ^#^
CTA spot sign	36 (53.7)	10 (8.7)	<**0.001**^#^

^#^Chi-squared test, with percentages in parentheses. ^*^Independent samples t-test or Mann–Whitney U-test; data reported are the mean ± standard deviation. Boldface indicates statistical significance. HE, hematoma expansion.

### Radiological characteristics and the spot sign model

Statistically significant differences between the HE and non-HE groups were observed in the initial volume, shape, swirl sign, blend sign, black hole sign, island sign, and CTA spot sign (*P* < 0.05). However, there was no significant disparity between the two groups in time from symptom onset to baseline CT (*P* > 0.05) (Table 1).

A random stratified sampling approach was employed to divide the data from the 182 patients into a training set (145 cases) and a testing set (37 cases). These sets were then submitted independently to univariate analysis, and no significant differences in radiological characteristics between them were found (all *P* > 0.05). In both the training set and the testing set, the HE group displayed larger initial volume and a higher likelihood of exhibiting the blend sign and the CTA spot sign (all *P* < 0.05). In the training set, irregular shape (*P* = 0.015), the swirl sign (*P* = 0.046), and the island sign (*P* = 0.042) were associated with hematoma enlargement. In the testing set, there was a significant difference between the two groups in terms of location of the hematoma (*P* = 0.038). However, when hematoma location and the black hole sign were examined within their respective sets, no statistically significant differences were found between the two groups within either the training set or the testing set (all *P* > 0.05). Detailed results are presented in Table 2.

**Table 2:** Comparison of radiological characteristics between the HE group and the non-HE group in the training and testing sets.

	**Training set (*****n** =* **145)**			**Testing set (*****n** =* **37)**			***p-*value**
	**HE (*****n** =* **53)**	**non-HE (*****n** =* **92)**	***p-*** **value**	**HE (*****n** =* **14)**	**non-HE (*****n** =* **23)**	***p-*** **value**	
Location (deep/lobe)	34/19	65/27	0.418^#^	5/9	17/6	**0.038** ^#^	0.311^#^
Intraventricular hemorrhage extension	11 (20.8)	16 (17.4)	0.616^#^	4 (28.6)	4 (17.4)	0.445^#^	0.679^#^
Initial volume (mL)	42.6 ± 25.7	26.6 ± 16.1	<0.001^*^	45.9 ± 22.1	31.1 ± 20.8	**0.048** ^*^	0.245^*^
Shape, irregular	30 (56.6)	33 (35.9)	**0.015** ^#^	8 (57.1)	9 (39.1)	0.328^#^	0.785^#^
Swirl sign	21 (39.6)	22 (23.9)	**0.046** ^#^	5 (35.7)	2 (8.7)	0.080^#^	0.192^#^
Blend sign	18 (34.0)	13 (14.1)	**0.005** ^#^	6 (42.9)	2 (8.7)	**0.035** ^#^	0.974^#^
Black hole sign	6 (11.3)	4 (4.3)	0.209^#^	3 (21.4)	1 (4.3)	0.142^#^	0.651^#^
Island sign	10 (18.9)	7 (7.6)	**0.042** ^#^	4 (28.6)	2 (8.7)	0.173^#^	0.648^#^
CTA spot sign	28 (52.8)	7 (7.6)	<**0.001**^#^	8 (57.1)	3 (13.0)	**0.008** ^#^	0.485^#^

^#^Chi-squared test, with percentages in parentheses. ^*^Independent samples t-test or Mann–Whitney U test; data reported are the mean ± standard deviation. Boldface test statistical significance. CTA, computed tomography angiography; HE, hematoma expansion.

A binary LR model was constructed to analyze the CTA spot sign as a predictor of HE. In the training set, the AUC, sensitivity, specificity, and accuracy were 0.726, 0.528, 0.924, and 0.779, respectively. In the testing set, the AUC, sensitivity, specificity, and accuracy were 0.720, 0.571, 0.870, and 0.757, respectively (Table 3).

**Table 3:** Predictive performance of three models for HE in the training and testing sets.

		**AUC (95% CI)**	**Sensitivity**	**Specificity**	**Accuracy**
Training set	Spot sign model	0.726 (0.646, 0.797)	0.528	0.924	0.779
	NCCT model	0.938 (0.886, 0.971)	0.849	0.924	0.897
	CTA model	0.904 (0.844, 0.947)	0.774	0.902	0.855
Testing set	Spot sign model	0.720 (0.549, 0.855)	0.571	0.870	0.757
	NCCT model	0.925 (0.790, 0.986)	0.786	0.913	0.865
	CTA model	0.873 (0.722, 0.959)	0.714	0.913	0.838

AUC, area under the curve; CI, confidence interval; CTA, computed tomography angiography; HE, hematoma expansion; NCCT, non-contrast computed tomography.

### Construction and validation of radiomics models

**Figure 3:**
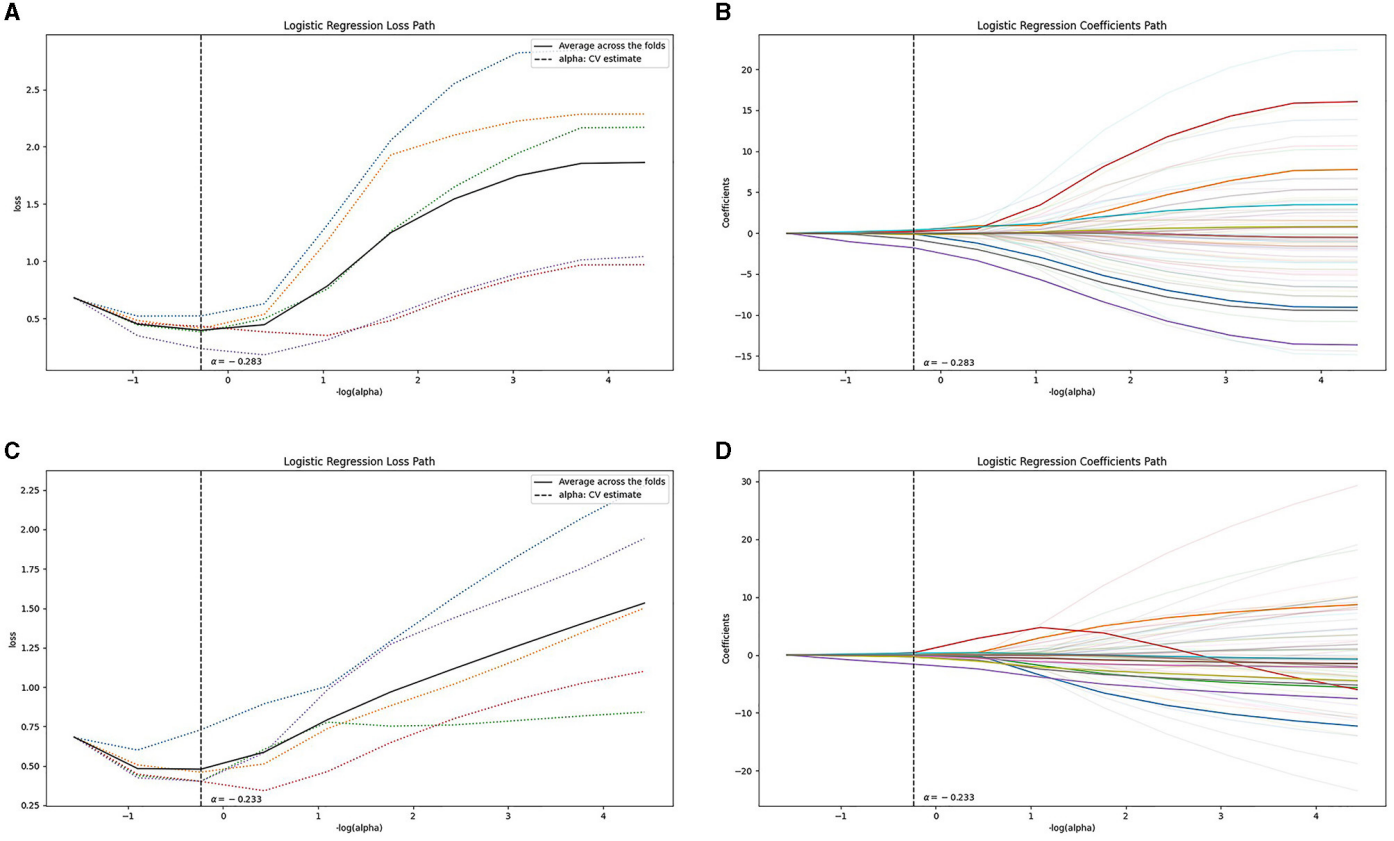
Feature selection using LASSO regression: the loss path of LASSO **(A, C)**, and the regression coefficients of LASSO **(B, D)**, for **(A, B)** the NCCT model and **(C, D)** the CTA model. CTA, computed tomography angiography; LASSO, least absolute shrinkage and selection operation; NCCT, non-contrast computed tomography.

**Figure 4:**
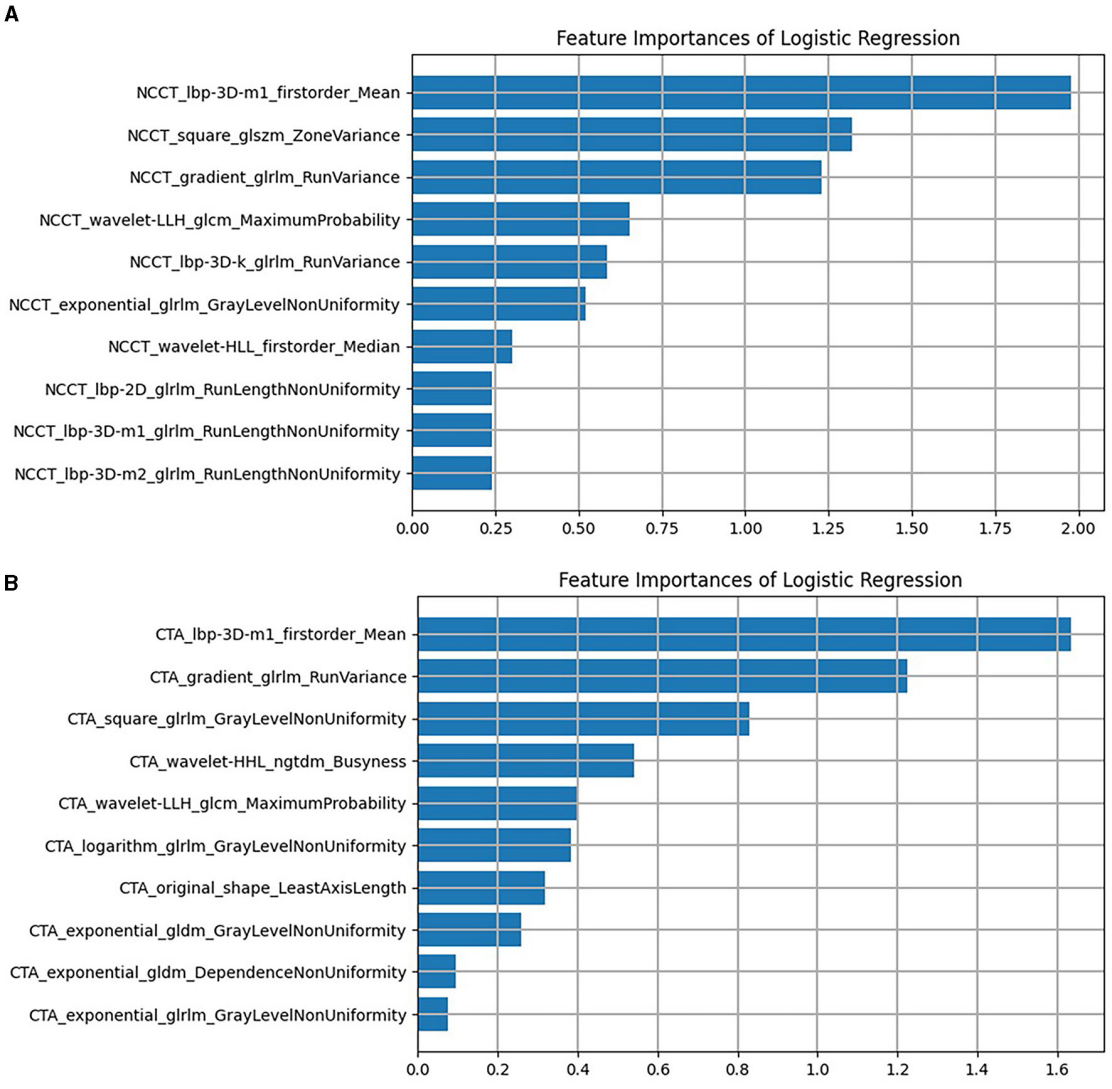
The remaining features of LASSO regression screening and the ranking of weight coefficients output by the corresponding LR classifier under **(A)** the NCCT model, **(B)** the CTA model. CTA, computed tomography angiography; LASSO, least absolute shrinkage and selection operation; LR, logistic regression; NCCT, non-contrast computed tomography.

Following the aforementioned screening methods, 10 optimal radiomics features were extracted from the NCCT and CTA images (Figure 3 illustrates feature selection using LASSO regression). The remaining features were employed to construct radiomics models in combination with the LR machine learning classifier, using a five-fold cross-validation approach (Figure 4). In the training set, the NCCT model achieved an AUC of 0.938, sensitivity of 0.849, specificity of 0.924, and accuracy of 0.897. Similarly, the CTA model yielded an AUC of 0.904, sensitivity of 0.774, specificity of 0.902, and accuracy of 0.855. In the testing set, the NCCT model achieved an AUC of 0.925, sensitivity of 0.786, specificity of 0.913, and accuracy of 0.865. Similarly, the CTA model resulted in an AUC of 0.873, sensitivity of 0.714, specificity of 0.913, and accuracy of 0.838 (Table 3).

### Comparison of the models on predictive performance

**Figure 5:**
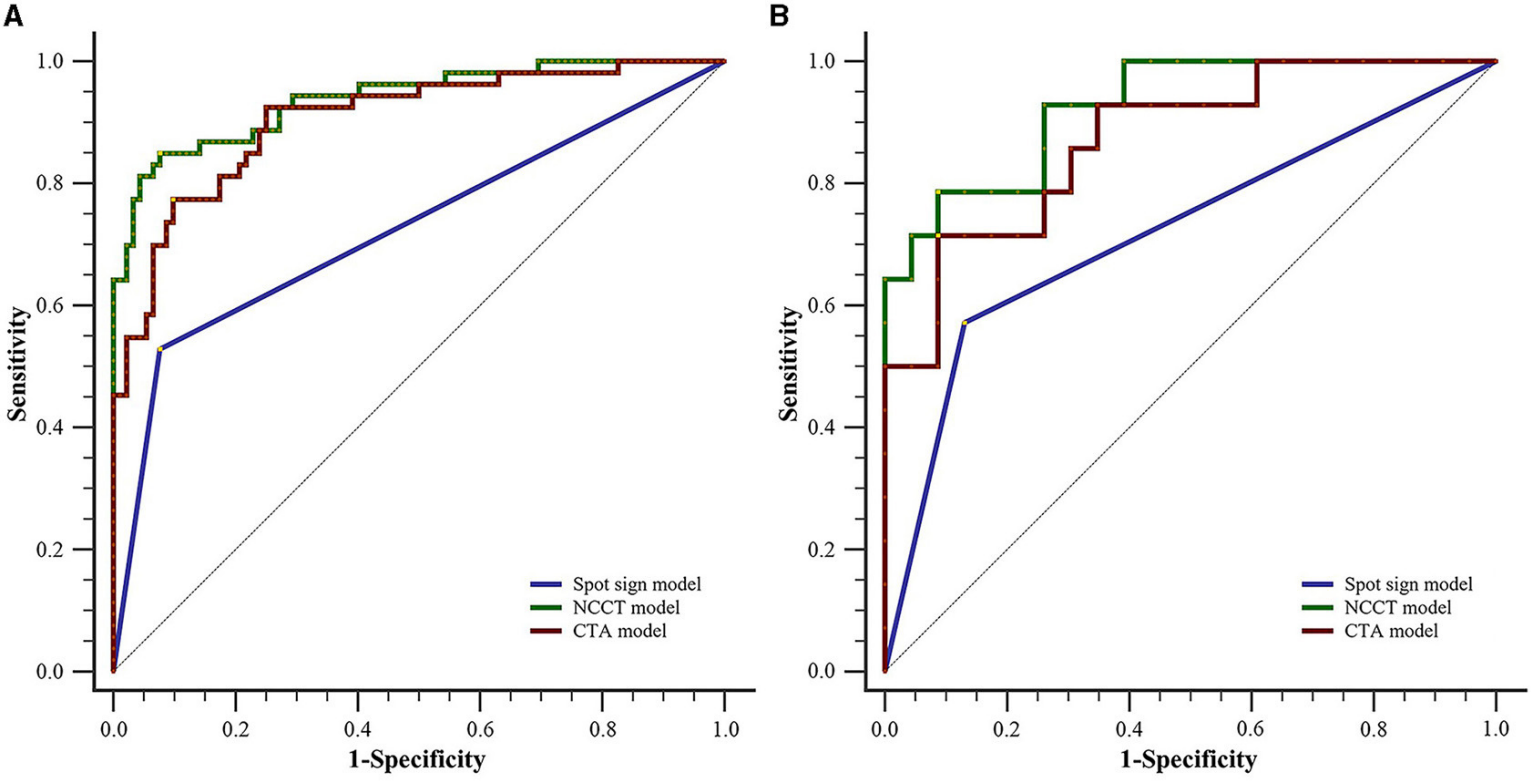
Comparison of ROC curves between the two radiomics models and the spot sign model for **(A)** the training set and **(B)** testing set. CTA, computed tomography angiography; NCCT, non-contrast computed tomography; ROC, receiver operating characteristic.

**Figure 6:**
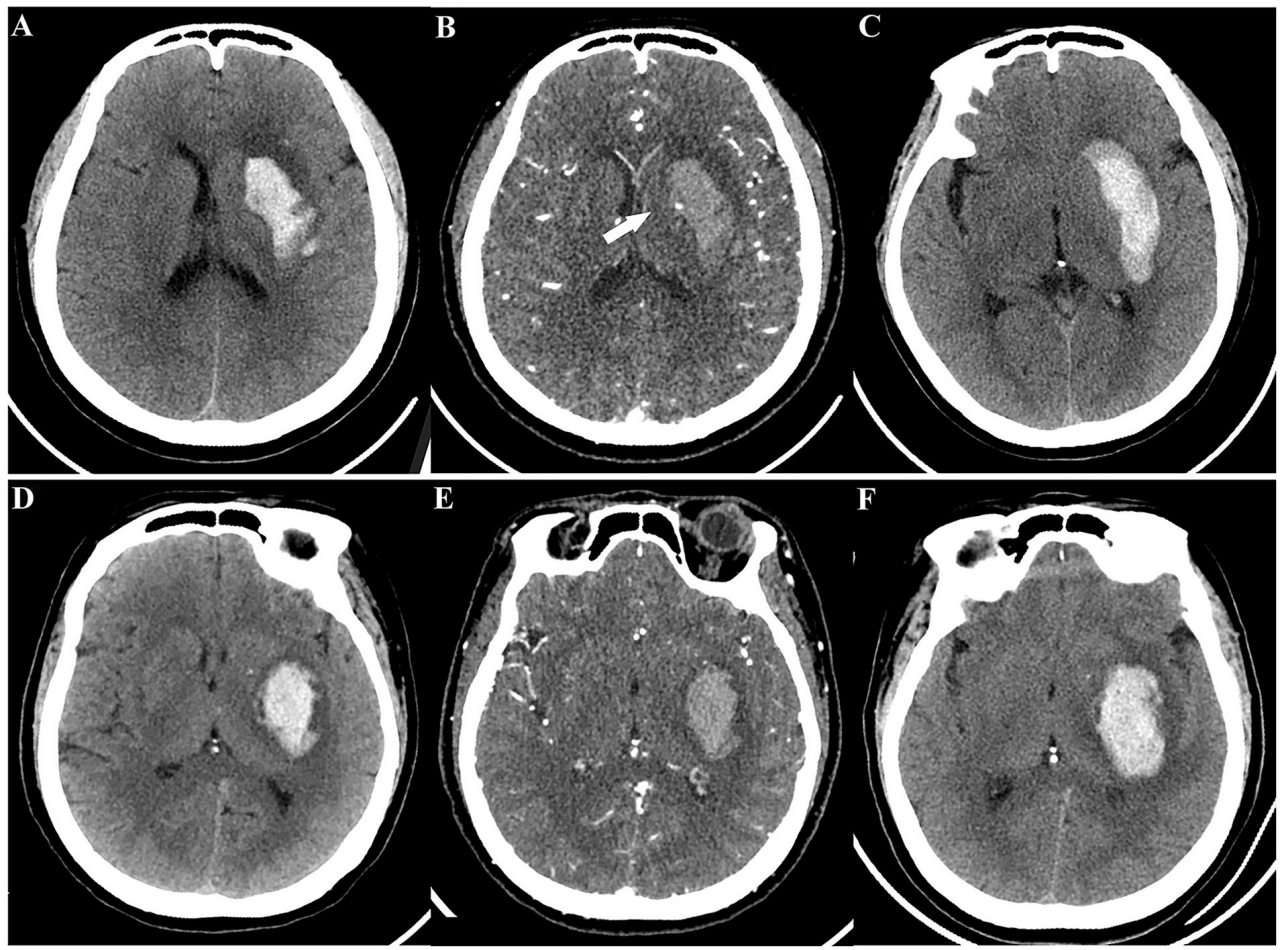
A comparison of two spontaneous intracerebral hemorrhage cases (with and without the spot sign) according to radiomics models. Case 1 with CTA spot sign, a 49-year-old woman: **(A)** initial NCCT; **(B)** CTA showed spot sign (white arrow); **(C)** follow-up CT revealed HE. Case 2 without CTA spot sign, a 55-year-old man: **(D)** initial NCCT; **(E)** CTA did not show spot sign; **(F)** follow-up CT still revealed HE. Both radiomics models provided successful predictions for these cases. HE, hematoma expansion; NCCT, non-contrast computed tomography; CTA, computed tomography angiography.

As illustrated in Table 4 and Figure 5, for the training set, both the NCCT model and the CTA model resulted in significantly larger AUCs compared to the spot sign model (all *P* < 0.001), while no statistically significant difference was observed between the NCCT model and the CTA model (*P* = 0.068). For the testing set, the NCCT model resulted in a larger AUC than the spot sign model (*P* = 0.041), while no significant difference was found between the NCCT model and the CTA model, or between the CTA model and the spot sign model (all *P* > 0.05). Figure 6 showcases a comparison of two SICH cases (with and without the spot sign). The radiomics models could successfully predict HE, even in cases where the spot sign was absent.

**Table 4:** Comparison of the three models on predictive performance for hematoma expansion.

	**Training set**	**Testing set**
NCCT model vs. CTA model	0.068	0.440
NCCT model vs. Spot sign model	<**0.001**	**0.041**
CTA model vs. Spot sign model	<**0.001**	0.182

Boldface indicates statistical significance. CTA, computed tomography angiography; NCCT, non-contrast computed tomography.

## Discussion

HE is a dynamic process influenced by active bleeding and serves as an important variable associated with clinical prognosis. In this study, we developed two radiomics models using NCCT and CTA images to anticipate HE and examined their performance in comparison to the CTA spot sign model. Our findings indicated that both radiomics models were effectively predictors of HE, demonstrating comparable performance. However, it is noteworthy that the NCCT radiomics model outperformed the traditional spot sign model in its predictive capabilities.

Initially, quantitative CT densitometry of hematoma through NCCT was utilized to predict ICH enlargement (22). However, this method provided limited information. Subsequently, dual-energy CT analysis of iodine concentration within the hematoma emerged as an improved approach for predicting HE. This analysis also led to the proposal of diffused leakage, indicating that the extravasation of contrast agents was not solely responsible for the aggregation observed within the hematoma (23). The advent of texture analysis and radiomics feature analysis in neuroimaging further contributed to the field. For instance, texture analysis parameters such as variance and uniformity demonstrated the ability to independently forecast HE following Laplacian of Gaussian operator filtering processing (24). Other researchers have subsequently demonstrated the predictive value of radiomics features for hematoma growth as well (17, 18).

Consistent with previous research findings (10, 25), our results reaffirmed the CTA spot sign as a well-established imaging marker for independently predicting HE in patients with ICH, demonstrating higher specificity than sensitivity. The appearance of the spot sign is believed to stem from contrast extravasation caused by ongoing bleeding from ruptured blood vessels (26). Meta-analyses have indicated that the sensitivity of the spot sign is ~53% (11), highlighting the fact that a significant portion of expanded hematomas may not exhibit this characteristic. Our study indicated that the radiomics models exhibited superior sensitivity to the spot sign.

In contrast to previous studies (17–19), our study utilized multimodal CT images and compared these with the spot sign. Prior research has demonstrated the superior predictive performance of the spot sign compared to NCCT signs (27), such as the blend sign. Hence, our initial assumption was that the CTA model would outperform the NCCT model. However, our findings contradicted this hypothesis. We speculate that these results may be attributed to the infiltration of contrast medium into hematomas during active bleeding, which occurs during CTA scans. Comparatively, the diffusion of contrast medium within the expanded hematoma weakens the disparities in CT values among each voxel, as well as the discrepancies in certain texture features. Consequently, the radiomics of the expanded hematoma exhibited similarities to those of stable hematomas in the CTA model.

This study has elucidated the value of radiomics as a predictive tool for HE, particularly in the significant number of patients who lack the spot sign. Additionally, radiomics models offered objectivity and convenience, unlike the spot sign, determination of which may be influenced by experiential bias. Integrating radiomics into clinical practice, specifically by utilizing NCCT-based radiomics models, could yield commendable predictive efficacy for HE, potentially reducing the need for unnecessary CTA examinations. This approach could consequently mitigate radiation exposure and minimize contrast agent usage.

Several limitations should be noted with respect to our study. First, certain patients were excluded due to either having undergone surgical treatment before follow-up CT or displaying motion artifacts. This exclusion could potentially have introduced biases into the results. Second, although manual segmentation of VOIs showed good reproducibility, automatic segmentation techniques may offer increased speed and accuracy, especially for larger sample sizes. Third, our sample size was limited due to the relatively small number of patients who underwent concurrent NCCT and CTA examinations, necessitating further validation in multi-center studies with larger cohorts.

## Conclusion

This study validated the predictive capability of radiomics models utilizing NCCT and CTA images for SICH expansion. Remarkably, our NCCT radiomics model exhibited superior performance compared to the spot sign model and was comparable to our CTA radiomics model. This has implications in terms of reducing the need for CTA examinations, thereby mitigating radiation exposure and contrast agent utilization. We firmly believe that radiomics analysis will play a crucial role in future clinical practice, aiding in treatment decisions for high-risk patients susceptible to HE.

## Data availability statement

The raw data supporting the conclusions of this article will be made available by the authors, without undue reservation.

## Ethics statement

The studies involving humans were approved by Ethics Committee of Northern Jiangsu People's Hospital. The studies were conducted in accordance with the local legislation and institutional requirements. Written informed consent for participation was not required from the participants or the participants' legal guardians/next of kin in accordance with the national legislation and institutional requirements.

## Author contributions

QL: Conceptualization, Data curation, Investigation, Methodology, Writing – original draft, Writing – review & editing. FL: Methodology, Writing – review & editing. HL: Software, Visualization, Writing – review & editing. YL: Data curation, Formal analysis, Methodology, Writing – review & editing. HC: Methodology, Writing – review & editing. WY: Formal analysis, Writing – review & editing. SD: Writing – review & editing. HZ: Conceptualization, Funding acquisition, Investigation, Methodology, Resources, Supervision, Writing – original draft, Writing – review & editing.
